# Malaria in under-five children: prevalence and multi-factor analysis of high-risk African countries

**DOI:** 10.1186/s12889-024-19206-1

**Published:** 2024-06-24

**Authors:** Jackline Vicent Mbishi, Suleiman Chombo, Pankras Luoga, Huda Jaffar Omary, Heavenlight A. Paulo, John Andrew, Isaac Yeboah Addo

**Affiliations:** 1 Muhimbili University of Health and Allied Sciences (MUHAS), Dar es Salaam, Tanzania; 2 University of Galway, Galway, Ireland; 3 Research Fellow and Tutor, Concord Clinical School, University of Sydney, Sydney, Australia; 4 Research Fellow, University of New South Wales, Sydney, Australia

**Keywords:** Malaria, mRDT, Parasitemia, Under-five children, sub-saharan Africa

## Abstract

**Background:**

Malaria remains a significant public health challenge in Sub-Saharan Africa (SSA), particularly affecting under-five (UN5) children. Despite global efforts to control the disease, its prevalence in high-risk African countries continues to be alarming, with records of substantial morbidity and mortality rates. Understanding the association of multiple childhood, maternal, and household factors with malaria prevalence, especially among vulnerable young populations, is crucial for effective intervention strategies.

**Objective:**

This study examines the prevalence of malaria among UN5 children in selected high-risk SSA countries and analyzes its association with various childhood, maternal, and household factors.

**Methods:**

Data from the Malaria Indicator Surveys (MIS) spanning from 2010 to 2023 were analyzed. A weighted sample of 35,624 UN5 children from seven countries in sub-Saharan Africa (SSA) known for high malaria prevalence was considered in the analyses. Descriptive statistics and modified Poisson regression analysis were used to assess the association of multiple factors with malaria prevalence. Stata version 15 software was used in analyzing the data and statistical significance was set at a 5% significance level.

**Results:**

The overall pooled prevalence of malaria among the studied population was 26.2%, with substantial country-specific variations observed. In terms of child factors, a child’s age was significantly associated with malaria prevalence (APR = 1.010, 95% CI: 1.007–1.012). Children of mothers with higher education levels (APR for higher education = 0.586, 95% CI: 0.425–0.806) and Fansidar uptake during pregnancy (APR = 0.731, 95% CI: 0.666–0.802) were associated with lower malaria risk. Children from middle-wealth (APR = 0.783, 95% CI: 0.706–0.869) and rich (APR = 0.499, 95% CI: 0.426–0.584) households had considerably lower malaria prevalence compared to those from poor households. Additionally, rural residency was associated with a higher risk of malaria compared to urban residency (APR = 1.545, 95% CI: 1.255–1.903).

**Conclusion:**

The study highlights a notable malaria prevalence among under-five (UN5) children in high-risk SSA countries, influenced significantly by factors such as maternal education, Fansidar uptake during pregnancy, socioeconomic status, and residency. These findings underscore the importance of targeted malaria prevention strategies that address these key determinants to effectively reduce the malaria burden in this vulnerable population.

## Introduction

Malaria is a potentially fatal acute disease resulting from infection by parasites transmitted through the bites of infected female *Anopheles* mosquitoes [1]. The disease stems from five distinct parasite species, however, *Plasmodium falciparum* are the primary contributors to the grave threat posed by the disease, particularly in sub-Saharan Africa (SSA) where the population is largely impacted [2, 3].

As of June 2023, only 40 countries had achieved a malaria-free status, reporting zero indigenous cases [4]. The majority of malaria incidents remain concentrated in Africa, particularly in the SSA region, constituting 88% of all reported cases [4]. Furthermore, malaria-related mortality showed a 50% reduction from the year 2000 to 2015, decreasing from around 30 to 15 deaths per 100,000 at-risk population [5]. This decline persisted, albeit more slowly, reaching 14 in 2019. However, it rose to 15.1 in 2020, followed by a marginal decrease to 14.8 in 2021 [5]. In the same year (2021), nearly all malaria-related deaths (96%) occurred in 29 countries, with four SSA African countries - Nigeria, the Democratic Republic of Congo, Niger, and Tanzania collectively contributing to over half of these deaths [5].

Report indicates that under five (UN5) children are particularly vulnerable to the transmission and impact of malaria largely due to their immature immune system and increased exposure to the malaria parasite [6, 7]. From the year 2000 to 2019, several global efforts in malaria control, including implementation of interventions such as insecticide-treated nets (ITNs), intermittent preventive treatment of malaria in infants (IPTi), and indoor residual spraying (IRS) have contributed to about 47% reduction in malaria incidence and mortality among UN5 children [8]. Despite these efforts, malaria-related deaths among children UN5, particularly, in SSA are still persistent with significant death rates in places like Nigeria and Tanzania, highlighting the ongoing need for more protective measures for this vulnerable group [8, 9].

Previous studies have identified several factors influencing malaria in children UN5 in SSA including usage of insecticide-treated nets (ITNs), environmental conditions, and access to healthcare services [9–12]. However, these studies had been largely country-specific and had not concentrated on the potentially connected and nuanced factors present across multiple high-risk countries. Additionally, there are changing dynamics in socioeconomic and demographic factors associated with malaria, especially in UN5 children [13–16], necessitating a continuous investigation into the prevalence and factors associated with the disease in this age cohort. Moreover, limited research has specifically focused on the influence of Fansidar uptake during pregnancy on malaria prevalence in UN5 in SSA. Considering these gaps, this study primarily investigated the prevalence of malaria in UN5 children in high-risk SSA countries using the most recent Malaria Indicator Surveys (MIS). The study further examined associations of multiple factors, including Fansidar uptake during pregnancy with the prevalence of malaria in the UN5 children. Findings from this study could guide the refinement of malaria intervention and control strategies in high-risk SSA countries and ultimately contribute to combating the deadly disease in this vulnerable demographic and geographic context. By identifying specific and current contributing risk factors, policymakers can develop tailored strategies to mitigate malaria transmission, improve healthcare practices, and enhance prevention efforts, thereby reducing the disease burden, particularly in high-risk SSA countries.

## Materials and methods

### Data sources

The most recent MIS data between 2010 and 2023 were analyzed. The MIS is funded by the U.S. President’s Malaria Initiative (PMI) and the Global Fund to fight AIDS, Tuberculosis, and Malaria [17]. The MIS data presented a unique opportunity to profile malaria infection in the selected countries, filling gaps in routine data often marked by unknown denominators and selection bias, as not all malaria cases are reported in health facilities [18–20]. Malaria testing involves a blood collection process via child’s finger or heel prick and use malaria Rapid Diagnostic Test (mRDT) kit to test for malaria. The rapid diagnostic test kits used in these surveys have been validated to ensure their accuracy and reliability [21]. Trained health technicians performed these tests in the field according to manufacturer instructions [19]. Each country involved in the survey adopted standard questionnaires, leading to variable data collection across surveys. The study’s methodological approach was comprehensive, with the Demographic and Health Surveys (DHS) program authorizing the use of the data after a thorough review of our concept note.

As shown in Table 1, this study is based on MIS datasets obtained from countries that have been previously identified as having a high prevalence of malaria, namely - Tanzania, Mozambique, Angola, Burkina Faso, Nigeria, Uganda, and Niger. Although the Democratic Republic of Congo (DRC) holds the status as one of the highest malaria-endemic countries in Africa, it was not included in this study because the relevant data for the country was unavailable in the MIS [22, 23].

**Table 1:** Selected countries by year of survey

Country	Year of survey
Tanzania	2022
Nigeria	2021
Niger	2021
Uganda	2018/2019
Burkina Faso	2017/2018
Mozambique	2018
Angola	2011

### Study population

The weighted sample size was 35,624 UN5 children (aged 6–59 months), who underwent malaria rapid tests across the seven SSA countries. The sample was weighted to ensure a precise representation of the target population in these countries. As recommended by the DHS, sample weights were calculated by dividing the household sample weight by 1,000,000 [24].

### Sampling procedure

The surveys employed a two-stage stratified cluster sampling method. Initially, clusters, typically a district or village serving as primary sampling units, were selected based on the population and housing census frameworks of the included countries. The second stage involved choosing households from each cluster using systematic random sampling. A detailed description of the MIS sampling design and data collection procedures can be found in each country’s DHS report [25–27].

### Study variables and measurements

#### Outcome variable

The dependent or outcome variable in this study was the malaria parasitological test results in children aged 6–59 months, identified through malaria rapid diagnostic tests. The variable was defined in a binary manner, with a positive malaria result coded as 1 and a negative result coded as 0.

#### Explanatory variables

Informed by previous literature [11, 12, 28, 29] ten explanatory variables were selected. The explanatory or independent variables were grouped into three categories: characteristics of the child (age and sex), characteristics of the mother (highest education level and Fansidar use during pregnancy), and household characteristics (wealth index, sex of the household head, age of the household head, bed net usage, and residence type). Detailed descriptions and categorization of the explanatory variables are presented in Table 2.

**Table 2:** Descriptions of the explanatory variables

Variable	Categorization
Child Characteristics	
Age	Age of the child was in months and measured as a continuous variable
Sex	Sex of the child was categorized as male and female
**Maternal characteristics**	
Highest education level	Mother’s highest education level was categorized as no education, primary education, secondary education, and higher education
Fansidar intake during pregnancy	This involved the question of whether a mother took *Sulfadoxine-Pyrimethamine (SP)*/Fansidar during pregnancy which was categorized as yes or no
**Household Characteristics**	
Bed net use	This referred to the question of whether children UN5 slept in a bed net the last night preceding the survey which was categorized into yes or no
Age	This variable involved the age of the household head in years which was categorized as 10–24, 25–34, 35–49, 50, and above
Sex	Sex of the head of the household was categorized into male and female
Wealth status	Wealth status of the household was categorized as poor, middle and rich
Residence	Type of residence of the household was categorized into rural and urban

### Data processing and analysis

The study used the appended weighted datasets from the seven SSA countries. The children MIS (KR) dataset, household MIS (PR) dataset and the individual (IR) datasets for each of the seven countries were first weighted and merged into a single country-specific dataset. Each country’s dataset had its unique ID, but shared similar column structures, making it feasible to append them (merging of datasets for all seven countries) into a single comprehensive dataset. The variables of interest, which had consistent naming conventions across countries, were thus integrated. We recorded the malaria prevalence variable (hml35) as ‘positive’ or ‘negative.’ Originally, this variable included other categories such as ‘not present,’ and ‘refused,’ which were all dropped to promote the accuracy of the findings. The analysis included only children living with the respondent (mother of a child), as it ensured a more accurate representation of the household’s health status and socio-economic conditions, which are critical factors in malaria prevalence.

The wealth index variable was originally divided into five categories: poorest, poorer, middle, richer, and richest. However, it was re-categorized into three groups for a more streamlined analysis: combining ‘poorest’ and ‘poorer’ as ‘poor’ category, ‘richer’ and ‘richest’ as ‘rich’ category, and retaining ‘middle’ as a separate third category. This re-categorization was aimed at capturing the broader economic strata of the population, facilitating a more generalized understanding of the economic impact on malaria prevalence. Furthermore, the age of the household head was categorized into four groups: 10–24 years, 25–34 years, 35–49 years, and 50 + years. This categorization was based on the typical life stages and responsibilities associated with these age ranges, which could influence household health practices and, consequently, malaria prevalence. Other variables were utilized as recorded in the MIS data.

The study’s initial analysis involved descriptive statistics, encompassing frequencies and percentages for categorical variables, and summary statistics like mean and standard deviation for continuous variables, to provide an overview and general understanding of the background characteristics of the study participants.

To assess the influence of various factors on malaria prevalence, both univariable and multivariable modified Poisson regression analyses were performed, considering the survey design. The analysis proceeded in two stages, initially using a univariable Poisson model to evaluate the impact of each covariate independently on malaria prevalence in UN5 children (6–59 months), and subsequently employing an adjusted modified Poisson model incorporating multiple covariates. Model diagnostic tests were performed for the fitted multivariable logistic model including an assessment of multicollinearity using the Generalized Variance Inflation Factor (GVIF) and no variables were correlated. For this reason, all variables were included in the adjusted model. All statistical decisions were made at the 5% significance level, and analyses were conducted using Stata version 15 software.

### Ethical considerations

The study used secondary data and therefore no separate ethical approval was required. However, access to the data was granted through an online request to the measure DHS program (http://www.dhsprogram.com). The data used in this study are publicly accessible and do not contain any personal identifiers.

## Results

### Weighted prevalence of malaria in the seven SSA countries

**Fig. 1:**
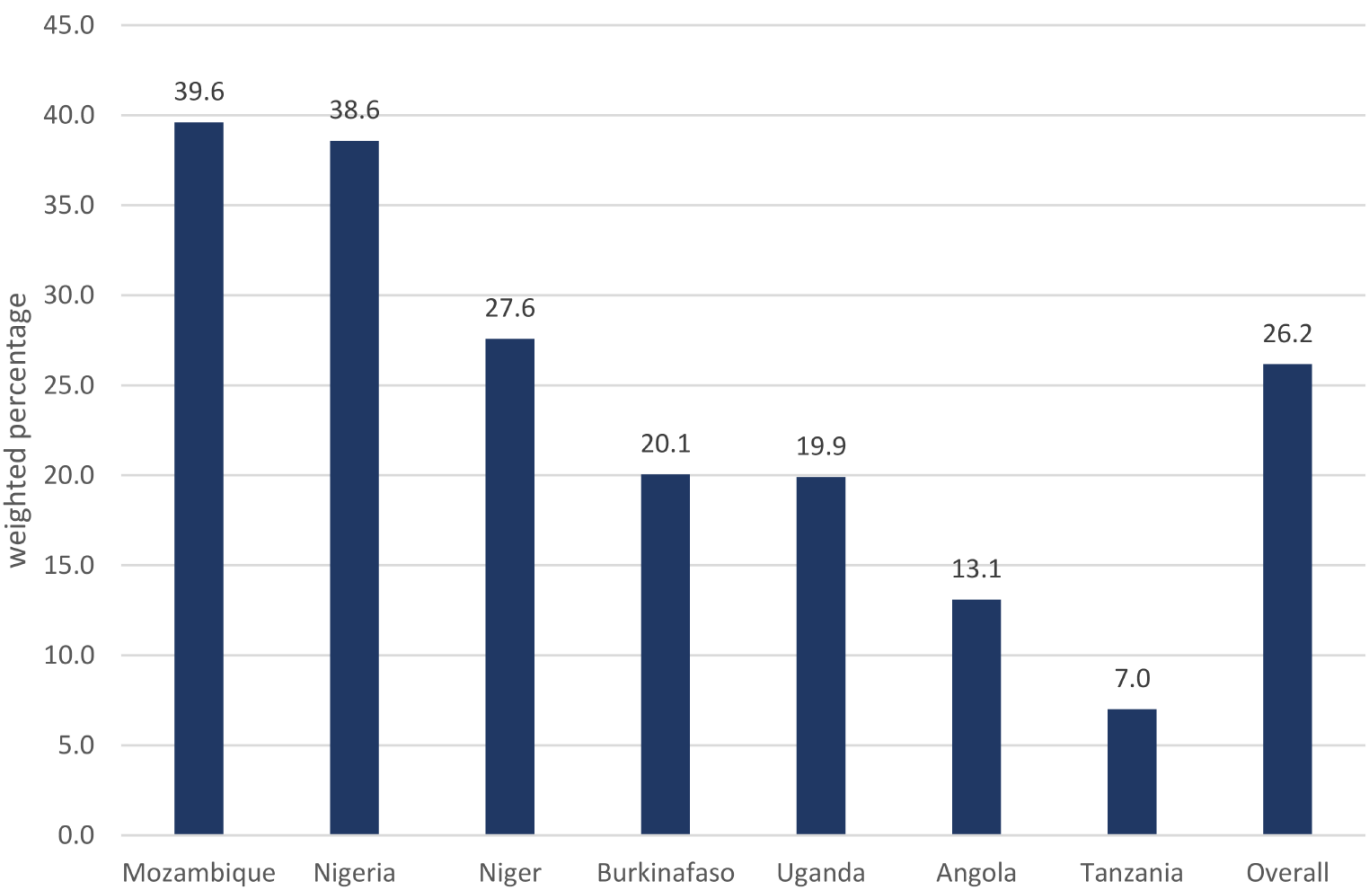
Weighted prevalence of malaria in seven high-risk African countries

Figure 1 displays the prevalence of malaria in the seven SSA countries. The overall pooled prevalence was 26.2%, with Mozambique having the highest malaria prevalence at 39.6%, and Tanzania sharing the lowest at 7.0%.

### Characteristics of the study participants

In this study, 35,264 weighted UN5 children (6–59 months) were tested for malaria, with 9,140 (26.2%) of them testing positive. Among these positive cases, the majority (52.2%) were male children, and 71.6% of their mothers reported taking Fansidar during pregnancy. A notable 57.5% of the children who tested positive had mothers with no formal education. The households of these malaria-positive children were predominantly headed by males (87.0%) and were largely characterized by poor wealth status (60.8%). Of all sampled households, the proportion of non-bed net usage was 50.4%. Additionally, 40.7% of all household heads were aged between 35 and 49 years while 88.4% resided in urban areas (See Table 3).

**Table 3:** Background characteristics of participants by positive malaria status

	Number of mRDT positive (%)*n* = 9,140	*N*
Child Characteristics		
*Age (months)*	Mean = 32, sd = 15.7	35,264
*Sex*		
Male	4,769 (52.2)	17,950
Female	4,371 (47.8)	17,314
**Mother characteristics**		
*Took sp/Fansidar during pregnancy*		
No	1,216 (28.4)	4,578
Yes	3,065 (71.6)	15,427
*Highest education level*		
No formal education	5,037 (57.5)	14,501
Primary	2,564 (29.3)	10,278
Secondary	1,015 (11.6)	6,288
Higher	138(1.6)	1,251
**Household characteristics**		
*Sex of the household head*		
Male	7,955 (87.0)	30,207
Female	1,184 (13.0)	5,057
*Wealth status*		
Poor	6,606 (60.8)	18,760
Middle	2,280 (21.0)	8,595
Rich	1,978 (18.2)	14,455
*Bed net use*		
No	5,458 (50.4)	20,406
Yes	5,365 (49.6)	21,145
*Age of the household head*		
10–24	536 (5.0)	2,007
25–34	2,710 (25.1)	11,239
35–49	4,396 (40.7)	17,135
50+	3,163(29.3)	11,203
*Residency*		
Urban	1,232 (11.6)	9,754
Rural	9,414 (88.4)	31,387

*N = Total number of children tested for malaria, n = Total tested positive for malaria and sd = standard deviation

### Modified poisson regression analysis of factors associated with malaria prevalence

Several factors were found to be significantly associated with malaria prevalence Table 4. Regarding child-related factors, each additional month of a child’s age was associated with a 1.01 increase in malaria prevalence (APR = 1.010, 95%CI: 1.007–1.012).

Maternal education level was a strong predictor of malaria prevalence. Children of mothers with primary, secondary, and higher education indicated lower risks (10%, 30%, and 41% respectively) compared to children of mothers with no formal education.

Children whose mothers had Fansidar uptake during pregnancy were associated with a 26.9% reduced risk of malaria (APR = 0.731, 95% CI: 0.666–0.802) compared to children whose mothers had no Fansidar uptake. Household characteristics were also associated with malaria prevalence. Children from middle-wealth and rich households had significantly lower risks of malaria (APR = 0.783 and 0.499, respectively) with respective 22% and 50% reduced risks compared to those from poor households. Likewise, place of residence was a significant factor with children living in rural areas showing a substantially higher risk of malaria compared to those in urban areas (APR = 1.545, 95% CI: 1.255–1.903). The results imply that children living in rural areas had 55% more risk of malaria compared to those living in urban areas.

Additionally, the age of the household head had a varied impact on malaria prevalence. Children from households with the head of household aged 25–34 and 35–49 years showed 16.7% (APR: 0.833; 95%CI: 0.724–0.957) and 15.4% (APR: 0.846; 95% CI:0.732–0.979) lower risk of malaria respectively, compared to those with the head of household aged 10–24 years. However, no significant difference was noted for children from households with heads aged 50 years and above.

Bed net usage among UN5 children indicated a 5.5% (APR = 0.945, 95% CI: 0.872,1.024) reduced risk of malaria compared to bed net non-usage. However, the results were not significant.

**Table 4:** Pooled bivariate and multivariable modified Poisson regression on the factors associated with malaria prevalence among seven African countries

Malaria prevalence	PR	[95% conf.interval]		APR	[95% conf.interval]	
Child characteristics						
*Age* (months)	1.012***	1.011	1.014	1.010***	1.007	1.012
*Sex of child*						
Male	1			1		
Female	0.950*	0.909	0.993	0.940	0.884	1.001
**Mother characteristics**						
*Educational level*						
no education	1			1		
Primary	0.718***	0.657	0.785	0.903*	0.815	0.999
Secondary	0.465***	0.419	0.516	0.695***	0.603	0.802
Higher	0.318***	0.255	0.396	0.586***	0.425	0.806
*Fansidar uptake*						
No	1			1		
Yes	0.748***	0.677	0.825	0.731***	0.666	0.802
**Household Characteristics**						
*Wealth status*						
Poor	1			1		
middle	0.728***	0.671	0.790	0.783***	0.706	0.869
Rich	0.387***	0.347	0.431	0.499***	0.426	0.584
*Age of household age*						
10–24	1			1		
25–34	0.884	0.783	0.998	0.833*	0.724	0.957
35–49	0.955	0.845	1.081	0.846*	0.732	0.979
50+	1.048	0.914	1.202	0.896	0.765	1.048
*Bed net use*						
No	1			1		
Yes	0.953	0.889	1.022	0.945	0.872	1.024
*Place of residence*						
Urban	1			1		
Rural	2.383***	2.036	2.790	1.545***	1.255	1.903

*P < 0.05, **P < 0.01, ***p < 0.001 which indicates statistical significance at a 5% significance level

PR-Prevalence Ratio

conf. interval- Confidence interval

APR- Adjusted Prevalence Ratio

## Discussion

Using the latest available MIS data, this study has examined malaria prevalence among UN5 children in high-risk SSA countries. Additionally, the study assessed the factors associated with malaria prevalence, including mother’s uptake of Fansidar during pregnancy. The analysis revealed that malaria is still a public health concern among UN5 children in the seven high-risk malaria-endemic countries as more than a quarter (26.2%) of the children tested positive for the disease in our sample. Our findings offer a more precise prevalence of malaria in this cohort compared to a recent systematic review, which reported a broad range of malaria prevalence, from 0.7 to 80.3%, with most falling between 11% and 50%, and a few studies reporting lower or higher prevalence rates [6] Furthermore, Mozambique had the highest prevalence of 39.6% while Tanzania had the lowest prevalence of 7%. This suggests that there may be varying levels of malaria burden even among the seven high-risk malaria endemic countries probably due to factors such as differences in climate and geography, socioeconomic status, healthcare system strength, vector control policies, and political commitments on malaria [30, 31].

As mentioned earlier, another significant focus of this study was to comprehensively assess the extent to which factors related to children, mothers, and households contribute to variations in malaria prevalence across the countries studied. Our data shows that factors such as a child’s age, mother’s Fansidar intake during pregnancy, mother’s education levels, household wealth index, age of the household head, and place of residence had significant associations with the prevalence of malaria among the UN5 children in the selected countries.

Specifically, the findings that malaria prevalence in UN5 children increased with age can be attributed to several reasons. Firstly, as children grow older, they tend to spend more time outdoors and may engage in activities that increase their exposure to mosquitoes [32]. Secondly, it is reasonable to suggest that younger children, especially those under one year old, maybe use bed nets more than their older counterparts, particularly when they sleep with their parents and when there is a limited number of bed nets in the household, thereby reducing their exposure to mosquitoes. The lower prevalence of malaria among children under one year may also be attributed to the acquisition of antibodies during pregnancy and breastfeeding, leading to a decreased prevalence compared to older children who are weaned from breastfeeding and receive less attention hence increased risk of exposure to mosquitoes [33]. These findings align with a study conducted in Soroti District, Uganda, which examined factors influencing the consistent use of bed nets [34], as well as a study analyzing malaria profiles in 11 sub-Saharan Africa [35].

The significant association found between the intake of Fansidar during pregnancy and protective effects against malaria infection in UN5 children is not particularly surprising as evidence shows that Fansidar uptake by mothers can help reduce the incidence of placental malaria [36, 37]. Thus, our finding is consistent with recent literature that emphasizes the role of Fansidar uptake during pregnancy in preventing malaria-related outcomes in children [23]. The insights derived from this study contribute to the growing body of evidence supporting the integration of antimalarial interventions, especially during pregnancy, as a crucial strategy in safeguarding the health of both mothers and their young children. These findings reinforce the call for sustained efforts to ensure widespread access to and utilization of Fansidar during pregnancy to further advance the goals of malaria prevention in vulnerable populations.

Furthermore, the association between higher levels of education in women and reduced odds of contracting malaria in UN5 children is unsurprising. Educated women are more likely to be aware of malaria control measures such as bed nets and indoor residual spraying compared to those without formal education. Education can also elevate household incomes and can influence women to secure higher-paying jobs which increases their affordability of healthcare services and information which in turn can increase their chances of understanding the usefulness of malaria prevention. These findings are consistent with outcomes in previous studies [38–40], echoing the positive impact of education on health outcomes. Therefore, we advocate for the prioritization of educational initiatives aimed at women, not only for the individual benefits they reap but for the broader societal gains in health and well-being.

The impact of the household wealth index on malaria infection among UN5 is widely documented and was also evident in the current study. As household income rises, the likelihood of malaria infection decreases, with children from middle and affluent families exhibiting lower odds of contracting malaria compared to their counterparts from impoverished families. This reality may be attributed to wealthier households implementing better housing improvements, thereby reducing exposure to mosquitoes. In contrast, economically disadvantaged households may lack such enhancements, leaving their members more vulnerable to mosquito exposure. Moreover, wealthier households are likely to be more educated, possess superior knowledge of malaria prevention measures, and improved access to healthcare services. This pattern aligns with the findings of [35, 40, 41]. Therefore, addressing socioeconomic disparities is imperative for comprehensive malaria control strategies. This approach should encompass not only housing improvements but also educational initiatives and improved healthcare access to mitigate the disproportionate burden borne by impoverished families.

The study findings indicate that children residing in rural households face higher odds of malaria prevalence compared to their urban counterparts. This discrepancy may be attributed to the limited access to various amenities in rural areas, including better housing conditions, educational opportunities, and healthcare access. These observations correspond with the findings of [38, 39] supporting the notion that rural environments are conducive to higher malaria transmission rates. The multifaceted challenges faced by rural communities collectively underscore the urgency of targeted interventions. Addressing these disparities and fortifying malaria control measures in rural areas are imperative steps toward mitigating the disproportionate burden of malaria borne by these communities.

The World Health Organization (WHO) advocates for the utilization of insecticide-treated nets (ITNs) as a critical element in malaria control and elimination strategies due to their high efficacy in preventing infections and reducing disease transmission. Ensuring widespread access to and use of ITNs within households and communities is paramount in curbing the incidence of malaria. Numerous studies have affirmed that households with several mosquito nets experience a lower likelihood of malaria infection. Moreover, the risk is further diminished in children residing in households equipped with insecticide-treated mosquito nets and in communities with high ITN utilization rates, as indicated by [40]. Interestingly, our study deviates from these findings, as bed net usage did not significantly reduce the odds of malaria in both crude and adjusted models. This discrepancy may be attributed to the inconsistent use of ITNs and the tendency for children to spend time outdoors at night, exposing them to the risk of malaria infection due to the esophagus-exophilic mosquito biting behavior, as reported by [42]; further emphasizing the need for comprehensive strategies that address not only bed net distribution but also consistent and proper utilization, particularly in populations with nocturnal outdoor activities. This unexpected outcome could also be attributed to the existence of unseen confounding variables within the dataset. This insight underscores the importance of context-specific considerations when implementing malaria control measures to enhance their effectiveness.

Based on the findings, we highly recommend extensive support from both governmental and non-governmental organizations to address the substantial risk of malaria among UN5 children in Africa. Targeted interventions should prioritize the following groups: older children, mothers with lower education, pregnant women, economically disadvantaged mothers, and mothers residing in rural areas. Focusing on malaria intervention programs on these specific groups is essential to achieve the maximum reduction in malaria prevalence. It is also important to draw lessons from successful experiences in other regions, such as China. By studying and adapting successful malaria control programs from China [43], SSA countries can enhance their efforts to combat malaria effectively. It is also crucial to develop community-based programs that involve a multifaceted approach to malaria prevention, including the distribution of bed nets, provision of antimalarial medications including Fansidar among pregnant women, and educational campaigns on preventive measures and control. Additionally, careful consideration should be given to avoid inadvertently promoting the overuse of antimalarial medications in malaria prevention efforts. By addressing the malaria prevalence problem in a targeted and comprehensive manner, SSA countries can make significant contributions in reducing the impact of malaria on UN5 children, ultimately improving the health and well-being of these vulnerable populations.

### Strengths and limitations of the study

The study possesses several notable strengths. Foremost, the utilization of large-scale datasets from MIS ensures a comprehensive analysis, offering a broad representation of populations across diverse regions within the SSA countries under investigation. The inclusion of multiple associated factors allows for a more nuanced understanding of the complex determinants influencing malaria positivity in these nations. This comprehensive approach aids in identifying potential multifaceted interactions between various factors, providing valuable insights into the intricate dynamics of malaria prevalence in the studied regions. Moreover, the study’s focus on multiple countries allows for comparative analyses, facilitating the identification of both common trends and country-specific variations, contributing to a more holistic view of malaria epidemiology in the seven SSA countries.

However, the study’s reliance on the mRDT as a diagnostic tool presents inherent limitations that affect the reliability and precision of the findings. Despite its promptness and suitability for resource-limited settings, the mRDT approach is susceptible to cross-reactivity with non-malarial infections or alternative antigens [44]. These inaccuracies have the potential to distort our understanding of malaria prevalence, especially in areas characterized by both high and low transmission rates. Such discrepancies could lead to either an overestimation or underestimation of true prevalence, affecting the precision of our findings.

Additionally, the use of cross-sectional data constrains our ability to infer causality. By capturing data at a singular point in time, we are limited in our capacity to delineate the directional relationships among the variables under study. This limitation is critical, as it impacts our ability to draw definitive conclusions about the causes and effects underlying the observed patterns of malaria transmission.

Moreover, the exclusion of vital environmental variables, due to limitations in data accessibility, narrows our comprehension of the transmission dynamics of malaria. Not including these key factors in our study might have resulted in an incomplete representation of the complex interactions between environmental conditions and malaria prevalence.

Given these considerations, our findings should be interpreted with caution. They underscore the imperative for further research aimed at overcoming these limitations, thereby enriching our understanding of malaria epidemiology in SSA. Such efforts are essential to refining our strategies for malaria control and prevention in the region.

## Conclusion

The study underscores malaria’s continued prevalence among UN5 children in high-risk SSA countries, with significant variations influenced by maternal, child, and household factors. Notably, Fansidar uptake during pregnancy emerges as a protective factor against malaria, emphasizing the importance of antimalarial interventions during pregnancy. The study’s findings advocate for targeted malaria control strategies that address the identified risk factors, including enhancing maternal education, improving socioeconomic conditions, and focusing on rural communities. While the study provides crucial insights into malaria prevalence, its reliance on mRDT and cross-sectional data, along with the exclusion of environmental variables, calls for cautious interpretation and further research. Addressing these limitations is essential for developing more effective malaria prevention and control measures, ultimately contributing to the reduction of malaria burden among vulnerable populations in sub-Saharan Africa.

## Data Availability

Data that support the findings of this study is available upon request from Demographic Health Survey (DHS) data. Link-https://www.dhsprogram.com/data/dataset_admin/login_main.cfm.
